# Chronic PARP-1 inhibition reduces carotid vessel remodeling and oxidative damage of the dorsal hippocampus in spontaneously hypertensive rats

**DOI:** 10.1371/journal.pone.0174401

**Published:** 2017-03-24

**Authors:** Krisztian Eros, Klara Magyar, Laszlo Deres, Arpad Skazel, Adam Riba, Zoltan Vamos, Tamas Kalai, Ferenc Gallyas, Balazs Sumegi, Kalman Toth, Robert Halmosi

**Affiliations:** 1 1st Department of Medicine, Clinical Centre, University of Pecs, Pecs, Baranya, Hungary; 2 Szentagothai Research Centre, University of Pecs, Pecs, Baranya, Hungary; 3 Department of Biochemistry and Medical Chemistry, Medical School, University of Pecs, Pecs, Baranya, Hungary; 4 Department of Pathophysiology and Gerontology, Medical School, University of Pecs, Pecs, Baranya, Hungary; 5 Department of Organic and Pharmacological Chemistry, Medical School, University of Pecs, Pecs, Baranya, Hungary; 6 MTA-PTE Nuclear and Mitochondrial Interactions Research Group, University of Pecs, Pecs, Baranya, Hungary; Max Delbruck Centrum fur Molekulare Medizin Berlin Buch, GERMANY

## Abstract

Vascular remodeling during chronic hypertension may impair the supply of tissues with oxygen, glucose and other compounds, potentially unleashing deleterious effects. In this study, we used Spontaneously Hypertensive Rats and normotensive Wistar-Kyoto rats with or without pharmacological inhibition of poly(ADP-ribose)polymerase-1 by an experimental compound L-2286, to evaluate carotid artery remodeling and consequent damage of neuronal tissue during hypertension. We observed elevated oxidative stress and profound thickening of the vascular wall with fibrotic tissue accumulation induced by elevated blood pressure. 32 weeks of L-2286 treatment attenuated these processes by modulating mitogen activated protein kinase phosphatase-1 cellular levels in carotid arteries. In hypertensive animals, vascular inflammation and endothelial dysfunction was observed by NF-κB nuclear accumulation and impaired vasodilation to acetylcholine, respectively. Pharmacological poly(ADP-ribose)polymerase-1 inhibition interfered in these processes and mitigated Apoptosis Inducing Factor dependent cell death events, thus improved structural and functional alterations of carotid arteries, without affecting blood pressure. Chronic poly(ADP-ribose)polymerase-1 inhibition protected neuronal tissue against oxidative damage, assessed by nitrotyrosine, 4-hydroxinonenal and 8-oxoguanosine immunohistochemistry in the area of Cornu ammonis 1 of the dorsal hippocampus in hypertensive rats. In this area, extensive pyramidal cell loss was also attenuated by treatment with lowered poly(ADP-ribose)polymer formation. It also preserved the structure of fissural arteries and attenuated perivascular white matter lesions and reactive astrogliosis in hypertensive rats. These data support the premise in which chronic poly(ADP-ribose)polymerase-1 inhibition has beneficial effects on hypertension related tissue damage both in vascular tissue and in the hippocampus by altering signaling events, reducing oxidative/nitrosative stress and inflammatory status, without lowering blood pressure.

## Introduction

Hypertension is one of the most important risk factors of cardiovascular diseases and also contributes to cognitive impairments via vascular alterations [1–3] and oxidative damage of neuronal tissue [4, 5]. Compromised cellular homeostasis of reactive oxygen (ROS) and nitrogen species (RNS) in vascular components are considered as causative factors in chronic hypertension and also mediate its detrimental effects on supplied tissues [6].

While various kinds of ROS feature distinct physiological regulatory functions in the vasculature, an imbalance in the production and elimination results in dysregulation of vascular tone [6–8]. Accordingly, a pro-oxidant state with inflammatory markers in vessels of human patients and animal models precedes the development of elevated blood pressure [7, 9]. It conveys detrimental effects, as accumulating ROS reacts with and therefore reduces the bioavailability of nitrogen monoxide (NO)—an important paracrine regulator of vascular tone -, by forming the highly reactive peroxynitrite (ONOO^-^) [10, 11]. It constitutes a feed-forward mechanism, where an imbalance in vasoconstrictor and dilator forces initiates remodeling of the stressed vasculature [6, 7]. In addition, an increased formation of ONOO^-^ in the vicinity of vascular endothelium activates the nuclear enzyme poly(ADP-ribose)polymerase-1 (PARP-1) [12–17] contributing to endothelial damage and dysfunction in various pathologies, including chronic hypertension [17]. In this manner, excess ROS production directly and also via PARP-1 activation, modulates activity of intracellular signaling routes and transcription factors [8, 18, 19]. Angiotensin 2 potentiated activation of mitogen activated protein kinases (MAPKs) regulates trophic responses and differentiation of cellular components in the vascular wall [20, 21] and at least partially mediate interstitial collagen accumulation [7, 22, 23]. PARP-1 is a co-regulator of nuclear factor kappa-light-chain-enhancer of activated B cells **(**NF-kB) during inflammatory response, and in this way, its activity contributes to additional ROS accumulation via immunological processes leading to deterioration of endothelial integrity and damage of surrounding tissues [24–27]. Excess PARP-1 activity has been shown to modulate stress related signaling routes and also initiate a caspase independent form of cell death, termed as parthanatos, in the scenario of myocardial ischemia/reperfusion damage [28] doxorubicin induced cardiac injury [29], hyperglycaemia related oxidative damage and endothelial dysfunction [30], acute septic shock [31] and chronic hypertension induced remodeling of rat aorta [32].

Hypertension induced alterations in the cerebral vasculature results in compromised blood supply of the highly energy demand organ. Additionally, inflammation and oxidative stress related endothelial damage and dysfunction leads to deterioration of blood-brain barrier integrity, propagating damage of neuronal tissue [6, 33–35]. Regarding these processes, the Spontaneously Hypertensive Rats (SHR) represents a chronic model characterized by perivascular lacunar infarcts and reactive astrogliosis [36, 37]. Notably, at the age of 6–8 months volume reduction and extensive cell loss have been described in the hippocampus of SHRs, a brain area highly sensitive to oxidative insults [38].

In a previous study, we successfully evaluated important molecular mechanisms of arterial remodeling regarding NF-ĸB signaling and MAPK members activity in the SHR model in relation to L-2286 treatment [32]. The current paper is aimed towards reinforcing these mechanisms at the level carotid arteries and to observe, whether modulating the remodeling process and aiding endothelium integrity is able to ease the genotoxic burden on supplied neuronal tissue. For these observations, the dorsal hippocampus was chosen as a model area to explore vasculature related perturbations in neuronal tissue and to quantify oxidative stress related pyramidal cell loss.

## Methods

### Animal model and noninvasive blood pressure measurement

10-week old male SHR rats, obtained from Charles River Laboratories (Budapest, Hungary) were randomly divided into two groups. One group received no treatment (SHR-C, *n* = 15), whereas the other group (SHR-L, *n* = 15) received 5 mg/kg/day 2-[(2-Piperidine-1-ylethyl)thio]quinazolin- 4(3H)-one (L-2286) [19, 32, 39–42], a water-soluble PARP inhibitor *ad libitum* for 32 weeks. As normotensive controls, age-matched Wistar-Kyoto (WKY) rats (Charles River Laboratories, Budapest, Hungary) were used with (WKY-L, *n* = 15) or without (WKY-C, *n* = 15) L-2286 treatment. Animals were caged individually and maintained on a 12h light/dark cycle at 24°C. L-2286 was dissolved in drinking water on the basis of preliminary data in reference to the volume of daily consumption. Prior to the beginning, and again at the end of the 32-week treatment period, ultrasound imaging was performed on each animal. Noninvasive blood pressure measurement was carried out every four weeks from the beginning of the study using the tail-cuff method (Hatteras SC 1000 Single Channel System) [43]. At the end of the study, animals were euthanized with an overdose of ketamine hydrochloride intraperitoneally and heparinized with sodium heparin (100 IU/rat i.p., Biochemie GmbH, Kundl, Austria). Carotid arteries were removed under an Olympus operation microscope and were fixed in buffered paraformaldehyde solution (4%). For brain histology, rats were anesthetized with an overdose of ketamine/xylazine then transcardially perfused with saline solution followed by buffered paraformaldehyde (4%). The investigation conforms to the Guide for the Care and Use of Laboratory Animals published by the US National Institutes of Health and was approved by the Animal Research Review Committee of the University of Pecs, Medical School (BA02/2000-2/2010).

### Carotid ultrasound examination

At baseline, all animals were examined utilizing ultrasound imaging to exclude rats with any abnormalities. Two-dimensional ultrasound was performed under inhalation anesthesia at the beginning of the study and on the day of sacrifice. Rats were lightly anesthetized with a mixture of 1.5% isoflurane and 98.5% oxygen. The necks and the upper side of the chest of animals were shaved, acoustic coupling gel was applied, and a warming pad was used to maintain normothermia. Intima-media thickness (IMT) of carotid arteries was measured using a VEVO 770 high-resolution ultrasound imaging system (VisualSonics, Toronto, Canada) equipped with a 40 MHz transducer.

### Isometric force measurement

The method was performed in accordance to a standard protocol using common carotid arterial (CCA) rings isolated from 4 rats each group. The contractile force was measured isometrically by using standard bath procedures [44]. Briefly, following ketamine/xylasine anesthesia, the carotid arteries were removed, quickly transferred to ice cold (4°C) oxygenated (95% O_2_ and 5% CO_2_) physiological Krebs solution (in mM: 119 NaCl, 4.7 KCl, 1.2 KH_2_PO_4_, 25 NaHCO_3_, 1.2 Mg_2_SO_4_, 11.1 glucose and 1.6 CaCl_2_.), and dissected into 5-mm rings. Each ring was positioned between two stainless steel wires (diameter 0.0394 mm) in a 5 ml organ bath of a Small Vessel Myograph (DMT 610M, Danish Myo Technology, Aarhus, Denmark). The normalization procedure was performed to obtain the basal tone to 1.0 g (13.34 mN), and artery segments were allowed to stabilize for 60 min prior to taking measurements. The software Myodaq 2.01 M610+ was used for data acquisition and display. The rings were pre-contracted and equilibrated for 60 min until a stable resting tension was acquired. Vasorelaxation is expressed as a percentage reduction of the steady-state tension, obtained with isotonic external 60 mM KCl. Cumulative response curves were obtained for sodium nitroprusside (SNP), acetylcholine (ACh), and KCl in the presence of endothelium. The bath solution was continuously oxygenated with a gas mixture of 95% O_2_ plus 5% CO_2_, and kept at 36.8°C (pH 7.4). Carotid rings were exposed to increasing doses of SNP (10^−9^ to 10^−5^ M), or ACh (10^−9^ to 10^−5^ M). Arterial rings showing relaxation to ACh of more than 30% were considered as endothelium intact. At the end of the experiments, the administration of 60 mM KCl was repeated to examine the viability of the carotid arteries. Each measurement was carried out on rings prepared from different rats.

### Immunohistochemistry and confocal laser scanning fluorescence microscopy

Carotid arteries and brain samples sent for immunohistochemical and immunofluorescence processing were fixed immediately following excision in a buffered paraformaldehyde solution (4%) overnight at 4 ^o^C. Five micrometer thick sections were cut from carotid arteries. From brain samples, 10 um coronal sections were taken at the position of bregma approx. (-4.3)–(-3.8) (Paxinos&Watson).

Fluorescence immunohistochemistry on carotid samples was performed for apoptosis inducing factor (AIF) (Cell Signaling Technology #4642, rabbit polyclonal, 1:100), NF-κB (Cell Signaling Technology #13586, rabbit monoclonal, 1:200) and MAP kinase phosphatase-1 (MKP-1) (Santa Cruz Biotechnology sc-370, rabbit polyclonal, 1:100). As secondary antibody, donkey-anti-rabbit antibody (Northern Lights, fluorochrome-labeled antibody, R&D Systems NL004, 1:200) was used. Sections were counterstained with Hoechst (Sigma) and examined using a confocal laser scan microscope (Olympus Fluoview 1000). Recording for Rhodamine Red™-X (excited with 557 nm Helium-Neon laser) was followed by recording for Hoechst with a 405 nm laser.

Carotid slices were stained with Masson’s trichrome to detect interstitial fibrosis and quantified with the NIH ImageJ analyzer system using the color deconvolution plugin to separate the blue collagen staining and measure its area coverage [45]. Carotid slices were also processed for nitrotyrosine (NT) (Millipore #06–284, rabbit polyclonal, 1:100) immunohistochemistry. Binding was visualized with biotinylated/HRP conjugated secondary antibody followed by the avidin-biotin-peroxidase detection system (PK-6200 Universal Vectastain ABC Elite Kit, Vector Laboratories, Burlingame, CA) using 3,3’-diaminobenzidine (DAB) as chromogen. Progress of the immunoreaction was monitored under a light microscope and the reaction was stopped by the removal of excess DAB with a gentle buffer wash.

Brain sections were processed for Cresyl violet or Periodic acid-Schiff (PAS) staining and also for immunohistochemistry with antibodies recognizing the following antigens: nitrotyrosine (NT) (Millipore #06–284, rabbit polyclonal, 1:100), 4-Hydroxynonenal (4-HNE) (a generous gift from Immunology and Biotechnology Department, Pecs, Hungary 1:200), poly(ADP-ribose)-polymer (PAR) (Abcam ab14459, mouse monoclonal, 1:400), 8-oxoguanine (8-OxG) (Abcam ab64548, mouse monoclonal, 1:500) and glial fibrillary acidic protein (GFAP) (1 Degree Bio #Z0334, rabbit polyclonal, 1:500). Immunolabeling was visualized by DAB as a chromogen. TUNEL test (R&D Systems, 4810-30-K) was conducted on embedded brain tissue samples accordance to the manufacturer’s protocol. All histological samples were acquired and examined by an investigator in blind fashion.

### Statistical analysis

Baseline comparisons between the strains were made by independent samples t-test. All the other analysis were conducted by a strain x treatment two-way ANOVA with 2 levels of each factor, followed by independent samples t-test in case of factor interactions in SPSS 21.0. All data are presented as mean±S.E.M. p<0.05 was considered statistically significant.

## Results

### L-2286 treatment attenuates structural remodeling of carotid artery walls, without lowering systolic blood pressure

**Fig 1:**
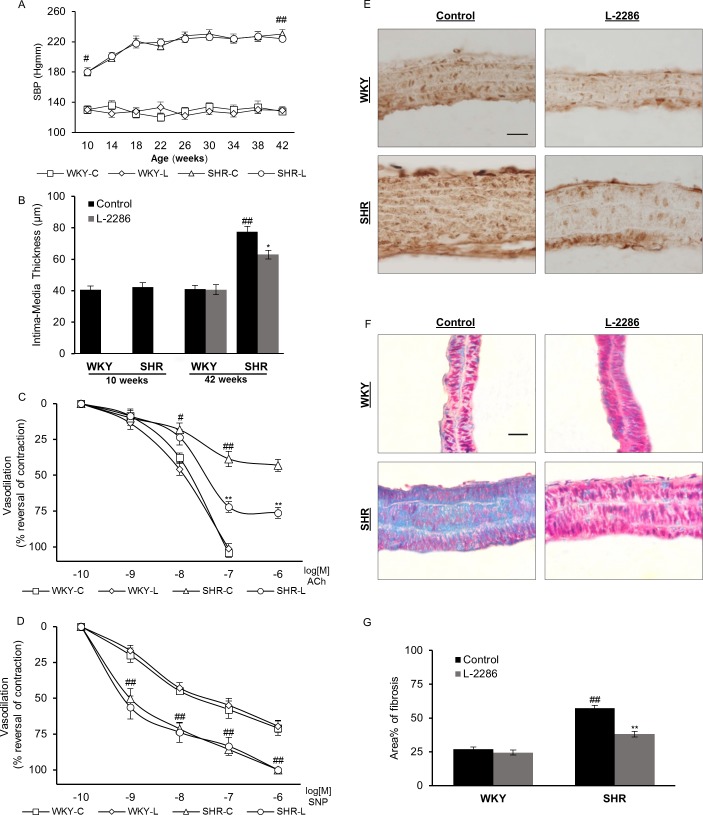
L-2286 treatment attenuated structural and functional remodeling of carotid arteries, without affecting SBP. (A) SBP of animals, measured every 4 weeks during the treatment period. (B) IMT of carotid arteries measured by ultrasound imaging at the beginning and at the end of the study. (C, D) Relaxation properties of isolated carotid artery rings against 60 mM KCl pre-contraction, in the presence of cumulative doses of (C) ACh and (D) SNP. (E) Representative micrographs of immunostaining for NT accumulation in carotid arteries (scale bar 30 μm). (F) Representative Masson’s trichrome stained micrographs (scale bar 30 μm) and quantification of (G) collagen accumulation in carotid artery walls. Data are presented as mean±S.E.M. ^#^p<0.05, ^##^p<0.01 vs. WKY-C; *p<0.05, **p<0.01 vs. respective controls.

Elevation of systolic blood pressure in the SHR strain was significant at the age of 10 weeks (130±5.4 Hgmm and 180±5.6 Hgmm for WKY and SHR strain, respectively, p<0.05) (Fig 1A). This difference was present throughout the treatment period (at the age of 42 weeks SBP was 128±4.8 Hgmm and 230±6.3 Hgmm in groups WKY-C and SHR-C respectively, p<0.01). L-2286 treatment did not exert any significant effect on SBP values compared to control groups (129±5.1 Hgmm, 224±3.4 Hgmm for WKY-L and SHR-L respectively, N.S.). Chronic hypertension induced arterial remodeling is characterized by wall thickening via expansion of vascular smooth muscle cells and an increase in interstitial fibrosis [23]. Therefore, we evaluated the IMT of carotid arteries. At baseline, the difference was not significant (40.68±2.36 μm, 42.38±2.64 μm for WKY and SHR, respectively, N.S.) (Fig 1B). By the age of 42 weeks, IMT of carotid arteries in the SHR-C group had almost doubled compared to normotensive animals. (41.1±2.4 μm, 77.5±3.42 μm for WKY-C and SHR-C respectively, p<0.01). While PARP inhibition attenuated this elevated blood pressure induced process (63±2.74 μm for SHR-L, p<0.05), it did not have any significant effect in normotensive animals regarding this parameter (40.6±3.15 μm for WKY-L, N.S.).

### L-2286 treatment had beneficial effect on the relaxation properties of isolated carotid arteries, in vitro

Active wall tension (mN/mm) of common carotid arteries was evoked by KCl (60 mM). This pharmacologically evoked contraction was similar to the maximal contractile force in each group. Dose-response curves to ACh and SNP (10^−9^ to 10^−5^ M range) were determined on CCA rings isolated from the control and L-2286 treated groups.

Cumulative response curves of CCA rings of SHR and WKY groups became distinguishable at the dose of 10^−7^ M ACh. (Fig 1C) While 10^−6^ M ACh completely reversed KCl induced wall tension of CCA rings isolated from normotensive animals, this reduction was only partial for hypertensive control rats (38.7±5.4%, p<0.01), without further improvement. 32 weeks of L-2286 treatment profoundly improved endothelium dependent relaxation capabilities of SHR-L CCA rings (72.2±3.7% and 76.3±3.8% for 10^−6^ and 10^−5^ M ACh respectively, p<0.01).

SNP induced vasorelaxation in the CCA rings of hypertensive control animals differed significantly from that of WKY-C animals (20±5%, 45.1±2%, 58±6.2%, 71.1±5% and 50.3±7%, 71.4±4%, 85.8±3%, 100.6±2.1% for WKY-C and SHR-C respectively, in the 10^−8^ to 10^−5^ M range of SNP, p<0.01) (Fig 1D). Cumulative response curves of WKY-L CCA rings was comparable with WKY-C animals (0%, 16.8±3.6%, 42.9±3.8%, 55±4.8, 69.6±4.2% for WKY-L, N.S.) L-2286 treatment slightly modulated relaxation capabilities of CCA rings isolated from hypertensive animals for 10^−8^ and 10^−7^ M SNP concentrations, although the difference was not statistically significant (0%, 56.4±8%, 73.8±7%, 83.5±6.3%, 101.8±3.2% for SHR-L, N.S.).

### Immunohistochemical and histological observations on carotid walls

Increased oxidative stress induced cellular dysfunction and cell loss leads to structural remodeling of vascular wall. We evaluated nitrosative damage in carotid arteries by NT staining (Fig 1E). An excess accumulation of ONOO^-^ byproducts was observed in the carotid walls of SHR-C animals, compared to normotensive controls. 32 weeks of L-2286 treatment attenuated this process in both strains. Structural remodeling due to chronic hypertension also includes an increased accumulation of fibrotic tissue in the vascular wall [46] quantified on Masson’s trichrome staining in the area of tunica media (Fig 1F). At the age of 42 weeks, area portion of fibrotic tissue was increased significantly in hypertensive animals (26.97±1.6%, 57.32±2.15% for WKY-C and SHR-C respectively, p<0.01) (Fig 1G). L-2286 treatment decreased the collagen content of carotid walls, this effect was more profound in hypertensive animals (24.5 ±1.9%, 38.1±2% for WKY-L and SHR-L respectively, p<0.01 vs. SHR-C).

**Fig 2:**
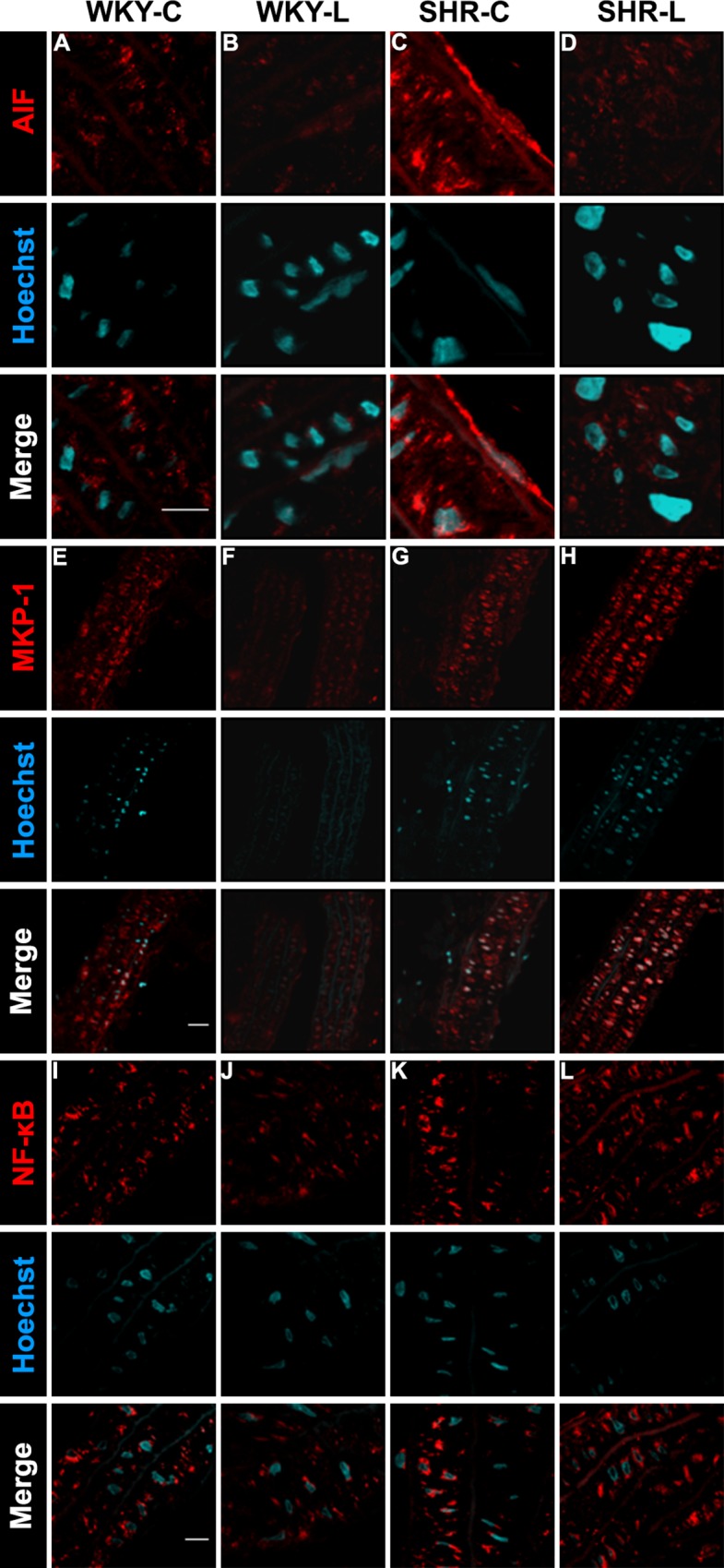
Representative micrographs of fluorescent staining for AIF and NF-kB cellular distribution and MKP-1 expression. (A-D) Nuclear translocation of AIF in carotid artery walls of (A) WKY-C, (B) WKY-L, (C) SHR-C and (D) SHR-L animals (scale bar: 10 μm). (E-F) Cellular level of MKP-1 in (E) WKY-C, (F) WKY-L, (G) SHR-C and (H) SHR-L animals (scale bar: 25 μm). (I-L) Subcellular distribution of NF-kB in (I) WKY-C, (J) WKY-L, (K) SHR-C and (L) SHR-L animals (scale bar: 10 μm).

**Fig 3:**
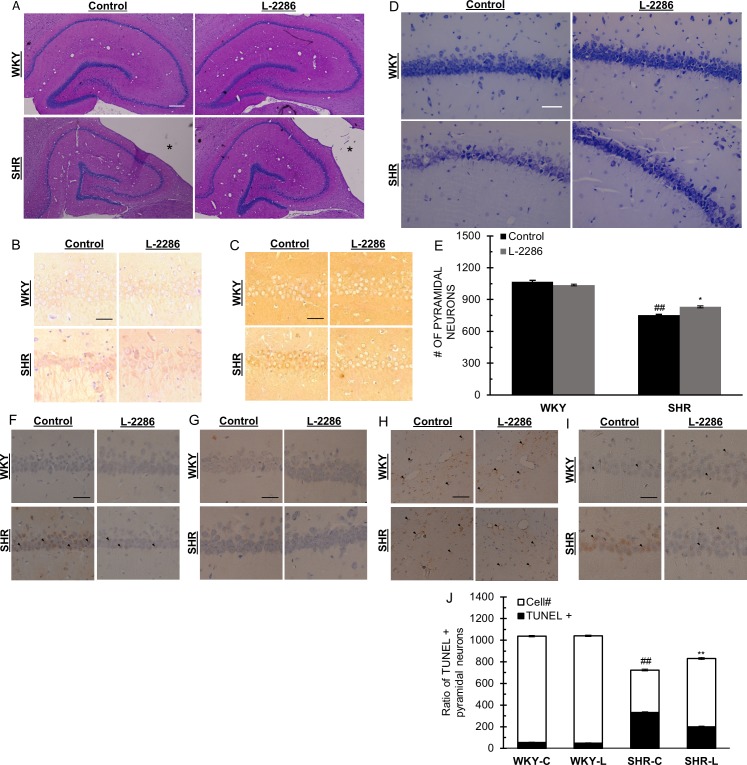
Pharmacological PARP-1 inhibition attenuated oxidative damage and cell loss in dorsal hippocampus. (A) PAS staining to evaluate structural alterations of the dorsal hippocampus and fissural vessels. *: Lateral cerebral ventriculus (scale bar: 200 μm). (B) NT and (C) HNE staining of CA1 regions for the evaluation of nitrosative damage and lipid peroxidation of pyramidal neurons in the dorsal hippocampus (scale bar 50 μm). (D) Representative micrographs of CA1 region of the dorsal hippocampus with Cresyl violet staining (scale bar 50 μm). (E) Pyramidal neuron number in the CA1 region of dorsal hippocampus. (F-H) Representative micrographs of immunostaining for (F) 8-oxG, (G) PAR and (H) GFAP (scale bar 50 μm) «: perivascular white matter damage. (I) TUNEL positive neurons and (J) numbers relative to pyramidal cells in the CA1 region of dorsal hippocampus (scale bar 50 μm). (F-H) ► points to positively stained cell. Data are presented as mean±S.E.M. ##p<0.01 vs. WKY-C; *p<0.05, **p<0.01 vs. SHRC.

One of the hallmarks of PARP-1 dependent cell death is the nuclear translocation of the mitochondrial inter-membrane space resident Apoptosis Inducing Factor (AIF), resulting in chromatin condensation and DNA fragmentation [47]. In carotid artery samples of normotensive groups (Fig 2A and 2B), AIF could only be found in extranuclear compartments. In the SHR-C group we observed translocation of AIF to the nucleus (Fig 2C), which was mitigated in the SHR-L group due to PARP-1 inhibition by L-2286 treatment (Fig 2D). Elevated cellular level of MKP-1 in carotid vessels of hypertensive control animals was observed (Fig 2G) relative to WKY-C samples (Fig 2E and 2F). The MKP-1 level was further increased by treatment in hypertensive animals (Fig 2H). In normotensive rats, NF-κB could be detected predominantly in the cytoplasm of cells (Fig 2I and 2J), while we observed enhanced nuclear translocation of this factor, related to chronic hypertension in SHR-C animals (Fig 3K). This process was diminished significantly by L-2286 treatment (Fig 2L).

### L-2286 treatment lowered oxidative damage in the area of dorsal hippocampus

We hypothesized that cellular protection via pharmacological inhibition of PARP-1 over activation is extendable to the neuronal tissue in chronic hypertensive animals. Evaluation of brains on macroscopic levels revealed a marked dilation of cerebral ventriculi in SHR animals. Increased pressure from the area of the 3^rd^ and lateral ventriculi distorted the hippocampal structure, seen on light microscopic preparations by the shortening of medial axis and the flattening of the crest of gyrus dentatus (Fig 3A). In chronic hypertensive animals, deformation of the transverse vessels of hippocampal fissure was observed with irregular lumen shapes. The structure of these vessels was more preserved in L-2286 treated SHR animals (Fig 3A).

To assess oxidative stress of neuronal tissue, histological samples were taken from the dorsal hippocampus. On NT stained sections (Fig 3B), accumulation of oxidative lipoprotein damage was apparent in the SHR-C group compared to normotensive controls. Also, lipid peroxidation byproducts visualized by 4-HNE immunohistochemistry (Fig 3C) demonstrated strong signal around pyramidal cells in this brain area of hypertensive control animals. L-2286 attenuated accumulation of these byproducts. Counting pyramidal neurons on Cresyl violet stained section (Fig 3D and 3E) in the area of Cornu Ammonis 1 (CA1) issued an extensive cell loss in SHR-C animals compared to normotensive controls (1067.12±15.19 and 753.25±6.19 for WKY-C and SHR-C respectively, p<0.01). L-2286 treatment attenuated this process in SHR-L animals in a significant manner (1037.75±7.23 and 831.25±8.98 for WKY-L and SHR-L respectively, p<0.05 vs. SHR-C). Augmented cellular oxidative and nitrosative stress results in accumulating damage of the DNA, leading to excess activation of nuclear PARP-1 [12]. Immunohistochemistry for the oxidative modification of guanine bases showed intense staining in the CA1 region of hypertensive control animals with a higher fraction of 8-oxoG positive cells compared to WKY-C rats (Fig 3F). Also, chronic hypertension led to augmented formation of poly(ADP-ribose)-polymer (PAR) in pyramidal neurons compared to normotensive controls (Fig 3G), with typical staining on the margins of nuclei. 32 weeks of L-2286 treatment attenuated oxidative DNA damage and PAR formation in hypertensive animals (Fig 3F and 3G). Elevated levels of oxidative stress resulted in excessive pyramidal cell loss in CA1 of SHR-C animals (Fig 3E), which was also observed by the higher portion of TUNEL positive cells in this region (WKY-C: 5.15±0.36% and SHR-C: 45.9±0.76%; p<0.01 vs. WKY-C) (Fig 3I and 3J). Chronic L-2286 treatment lowered the incidence of cell death of pyramidal neurons (WKY-L: 4.68±0.25% and SHR-L: 23.76±0.6%; p<0.01 vs SHR-C). A vasogenic perturbative environment of neuronal tissue and accentuated cell loss lead to activation of astrocytes. Reactive astrogliosis was visualized by GFAP immunohistochemistry (Fig 3H). No profound difference was found in the number of astrocytes between the strains at the area of hippocampal fissure, although alteration in their size as a sign of hypertrophy and a pronounced perivascular immunoreactivity was apparent in chronic hypertensive animals. 32 weeks of treatment by L-2286 reduced the numbers of activated astroglia in both strains with marginal effect on their reactive hypertrophy in SHR animals. The perivascular presence of reactive astrocytes was attenuated by applied treatment.

## Discussion

Hypertension in brain enhances oxidative stress, via activation of MAP kinases and cyclooxygenase (COX), elevated NO production and increased expression of Nox-2 (NADPH oxidase) [48, 49]. These processes lead to microglia activation, neuroinflammation and cell death [50]. Here we raise the possibility in which inhibition of PARP-1 could be beneficial in this scenario and this hypothesis was tested in SHR animals on the hypertension induced oxidative damage of carotid vessels and neuronal tissue. We observed structural remodeling of carotid arteries in hypertensive animals, characterized by a marked thickening of vascular wall (Fig 1B) and fibrotic tissue accumulation (Fig 1F and 1G). These processes, with an elevated inflammatory status may contribute to the vasomotor alterations seen by dilation properties of CCA rings (Fig 1C and 1D) in the SHR groups. Also, augmented oxidative stress of carotid walls (Fig 1E) and dorsal hippocampus (Fig 3B and 3C) was apparent in animals with chronic hypertension, underlined by the accumulation of nitrogen peroxide byproducts in these tissues.

The latter process results in diminished NO bioavailability [8, 51] and also contributes to the over-activation of PARP-1 via oxidants induced damage of the DNA [12, 26, 47, 52]. Excess formation of poly(ADP-ribose) polymers (PAR) alters nuclear NAD^+^ metabolism, originally considered as the main initiator of the caspase-independent form of cell death by cellular metabolic instability and a further boost in ROS production via mitochondrial dysfunction [13, 53, 54]. In our study, long term pharmacological PARP-1 inhibition by L-2286 treatment attenuated levels of oxidative biomarkers in carotid arteries (Fig 1E) and the dorsal hippocampus (Fig 3B and 3C) without having any significant effect on SBP (Fig 1A), as a causative factor. Based on previous results, the achieved protection is at least partially accountable for maintaining proper mitochondrial function, via aiding the function of respiratory chain complexes and preserves outer membrane integrity, therefore, lowered ROS production in these tissues are likely, due to a secondary process to pharmacological blockade of excess PARP-1 activation [28, 55, 56]. In addition to the NAD^+^ preserving effects, it prevents nucleus-to-mitochondria death signaling [47] and also interferes with stress-related biases in intracellular signaling routes [55, 57]. Amongst these, activation of JNK is considered to be a key event in the process of parthanatos via mitochondria damaging properties resulting in AIF release [47, 52, 58–60]. In our study, nuclear translocation of AIF was observed in control hypertensive animals (Fig 2C), indicating PARP-1 over activation initiated cell death events [47]. This process was mitigated by PARP-1 inhibition via L-2286 treatment in SHR animals (Fig 2D).

During chronic hypertension, oxidative stress leads to the activation of members of the MAPK system [8, 61]. Activated by growth factors and mechanical stretch, these kinases mediate maladaptive changes of vessels due to altered hemodynamic parameters, initiating transcriptional profiles leading to trophic responses and migration of vascular components [22, 62, 63], and also propagating accumulation of fibrotic tissue in the vascular wall [22, 32, 64]. Our group previously reported that prevention of PARP-1 enzymatic activity by genetic [65] or pharmacological means [32] elevated cellular levels of MKP-1, this way attenuating activity of MAPK members via dephosphorylation. Elevated MKP-1 expression due to PARP-1 inhibitor treatment was also observed in the current study by immunofluorescence (Fig 2F and 2H), which may form the molecular basis of attenuated thickening and lowered collagen content of carotid walls observed in our study in treated hypertensive animals.

Ultrastructural observation of SHR aorta in a previous study featured profound fibrotic tissue accumulation in the sub-endothelial space, where collagen bundles protruded into the vascular lumen disrupting the integrity of the endothelium [32]. Compromised barrier function by injury of the intimal layer and necrotic cell loss propagates inflammation of the vascular wall reflected by nuclear translocation of NF-κB in control SHRs carotid arteries (Fig 2K). During genotoxic stress, PARP-1 is involved in the regulation of NF-κB activation and nuclear trafficking [26, 66–69], and in this way their activity in concert results in upregulation of adhesion molecules and leukocyte invasion, which furthers ROS production via immunological processes [12, 24, 51, 70, 71] contributing to the evolution of an inflammatory vascular phenotype [25]. Pharmacological inhibition of PARP-1 resulted in lowered nuclear accumulation of NF-κB in our study (Fig 2L). Additionally, endothelial dysfunction of SHR-C carotid arteries was underscored by impaired relaxation properties in the presence of ACh (Fig 1C). Although the hyper-reactivity of SHR vessels to SNP was not affected by L-2286 treatment in a significant manner (Fig 1D), the above results indicate that endothelial NO production and response may be impaired in SHR animals, resembling previous observations in this model regarding the role of PARP-1 activation in the evolution of endothelial dysfunction [17]. A growing body of data demonstrates intermittent or long term modulation of PARP-1 activity attenuates the severity of endothelial dysfunction and even capable to reverse these processes [15–17, 72, 73].

Brains of SHR animals show well-described pathologies related to chronic hypertension, which make these animals a feasible model for studying target organ damages [36, 74]. A vasogenic oedema related dilatation of the cerebral ventriculi was observed [75]. This process seems to be independent of arterial pressure [76] and may relate to the deteriorated barrier function of the cerebrovascular endothelial layer induced by immunological processes and accumulating oxidative damage [33, 75, 77]. Increased pressure from the 3^rd^ and lateral ventriculi exerts mechanical stress on the area of dorsal hippocampus, distorting its structure (Fig 3A) and may partially relate to cell loss (Fig 3D and 3E) and volume reduction observed in this brain area [36, 38]. Also, hypertension induced structural remodeling of main arteries is also present in the cerebrovascular system of SHR animals, in connection with microvascular changes [2, 36, 37]. It compromises proper oxygen and nutrient supply of neuronal tissue, creating an ischaemic environment [74, 78]. Additionally, on a genetic background, SHR animals have impaired resistance against cerebral ischaemic insults early in life [79], predisposing this strain to augmented tissue damage in chronic hypertension. Perivascular white matter damage, lacunar infarcts are common in SHRs connected to altered cerebrovascular system and distorted endothelial function [37]. Irregular lumen shapes and an increased perivascular space of fissural arteries (Fig 3A and 3H) supplying hippocampal cortices were observed in control SHRs, related to chronic hypertension. Long term L-2286 treatment aided preserving structure of vessels in this area. Compromised barrier function and perturbations in the supply of neuronal tissue leads to the activation of astroglia population evident in young SHR animals by the elevated expression of GFAP in different brain areas, compared to their normotensive counterparts [36, 38, 74]. Regarding this process, in our study we found an increase in the average size of reactive astrocita in the hippocampal fissure of SHR-C animals (Fig 3H), without hyperplasic differences between the strains. Also, an altered vascular structure and permeability was delineated by stronger perivascular GFAP immunoreactivity and the presence of lacunar white matter damage around transverse vessels of hippocampal fissure in control hypertensive animals (Fig 3H). PARP-1 inhibition by L-2286 treatment apparently reduced the numbers of activated astrocytes in both strains with lowered perivascular accumulation in treated hypertensive rats. Reactive astrogliosis shows age dependent regional differences in the hippocampus of SHR animals, also the effect of PARP-1 inhibition induced ambiguous effects on different subpopulations in a study applying acute stress [80], related to PARP-1 activation. In our study, observations on activated astrocytes were restricted to the vasculature related alterations in hippocampal fissure. Our results indicate, applied treatment lowered the requirement for perivascular astroglial activity within this brain area. The reduced number of activated astrocytes by PARP-1 inhibition in normotensive animals raises the possibility of a molecular interference regarding astroglia activation [27, 69], which is not assessed in this study.

Ischaemic environment favors accumulation of ROS in neuronal tissue [81, 82] evaluated by immunohistochemistry in this study. A strong peri-somatic staining of 4-HNE was observable in the CA1 region of control hypertensive animals (Fig 3C), showing peroxidation of membrane lipids, which compromises cellular functions, propagating eventual death [83, 84] of pyramidal neurons. Also, as in carotid arteries, an increased level of nitrotyrosine adducts was evident in dorsal hippocampus of SHR-C animals (Fig 3B). Pharmacological inhibition of PARP-1 attenuated this process. Elevated nitrosative stress in this area also contributes to the accumulation of DNA damage by oxidative base modifications [52] assessed by 8-oxG immunohistochemistry (Fig 3F). These processes resulted in excess activation of PARP-1 enzyme in CA1 pyramidal neurons of control hypertensive animals (Fig 3G), leading to PARP-1 dependent cell loss. Accordingly, the accumulation of TUNEL positive neurons compared to normotensive control animals referring to apoptotic/necrotic processes was evident in SHR-C hippocampus (Fig 3I and 3J), attenuated by L-2286 treatment.

Thirty-two weeks of pharmacological PARP-1 inhibition by L-2286 treatment attenuated hypertension induced structural and functional alterations of carotid arteries by a lowered level of oxidative damage and an interference with stress related inflammatory and cell death propagating signaling events. This way without a significant effect on systolic blood pressure, pharmacological inhibition of excess PARP-1 activity at least partially helped to reduce stress on the supplied neuronal tissue and exerted additional protection against PARP-1 related pyramidal cell loss in the highly sensitive hippocampus of treated SHRs.

Given the long term and the applied number of animals in the study, comparisons to other PARP inhibitor compounds were not included. The authors are aware it may limit the current study regarding potential off-targets and the generalizability of conclusions.
